# Supplementary material for: Clinical and survival differences during separate COVID-19 surges: Investigating the impact of the Sars-CoV-2 alpha variant in critical care patients
Source: PLoS One. 2022 Jul 1;17(7):e0269244. doi: 10.1371/journal.pone.0269244 (PMC9249170; doi:10.1371/journal.pone.0269244)
Supplement: Attachment — Submitted filename: PLOS ONE - Responses to Reviewers - R2 -final.docx [file pone.0269244.s003.docx]

**Responses to Reviewers**

We would like to thank the editor for the opportunity to respond to the reviewers’ comments and are very grateful to the reviewers for their time in evaluating our paper. We have provided responses below to all of the concerns raised.

**Editor's comments:**

**Comment 1*:*** *‘Please find the reviewer's 2 comments for the remaining revision.’*

**Response 1:** We have responded to all of reviewer’s 2 comennts for the remaining revision

**Comment 2:** *‘Please prepare the tables per the standard "AMA Manual of Style".*

**Response 2:** We have prepared the tables as per the requested standard

**Comment 3:** *‘There is strong evidence denoting the importance of comorbidities on the survival of patients with COVID-19, such as cardiovascular disease (*[*https://academic.oup.com/eurjpc/article/28/14/1599/6145858*](https://academic.oup.com/eurjpc/article/28/14/1599/6145858)*), cancer (*[*https://brieflands.com/articles/ijcm-110907.html*](https://brieflands.com/articles/ijcm-110907.html)*,* [*https://www.ncbi.nlm.nih.gov/pmc/articles/PMC8184167/*](https://www.ncbi.nlm.nih.gov/pmc/articles/PMC8184167/)*), chronic kidney disease (*[*https://journals.plos.org/plosone/article?id=10.1371/journal.pone.0254525#:~:text=The%20crude%20mortality%20rate%20among,19%20in%20the%20PIRP%20cohort*](https://journals.plos.org/plosone/article?id=10.1371/journal.pone.0254525#:~:text=The%20crude%20mortality%20rate%20among,19%20in%20the%20PIRP%20cohort)*.), among others. I found that the authors have not evaluated this factor. I suggest considering this factor in the analysis; if the data are not available, please address it in the limitations by using the mentioned references’*

**Response 3:** Thank you for this comment and we have made the following change to our discussion and limitations:

*‘Thirdly, whilst we considered the covariates to include in our analysis, we did not account for other co-morbidities such as cardiovascular disease,^19^ cancer^20^ and chronic kidney disease,^21^ all of which have been associated with worse outcomes in COVID-19.’*

**Reviewer #2**

We thank the reviewer for his/her time and detailed, helpful comments and below are point-by-point responses to each of these. We have implemented the majority of the suggestions made by this reviewer, and provided explanations where we do not agree.

**Comment 1:** ‘*Though the authors have improved the manuscript draft; There are still few confounding variables which could have impacted the outcome in second wave that needs to be evaluated and mentioned in the limitations. (e.g., CT score, day of presentation)*’

**Response 1:** We thank the reviewer for this comment and have made the following changes to the discussion:

*Fourthly, scoring systems based upon computer tomography (CT) have been used to characterize COVID-19 severity and outcomes (e.g. requirement of mechanical ventilation/mortality) in patients, particularly on presentation to hospital.^22^ CT scores were not included in this study as all patients were already intubated and ventilated. Furthermore, we are unable to comment if ventilatory data at time of intubation correlated with CT score as most patients had CT scans when clinically stable to undertake transfer to radiology departments, rather than specifically at the onset of mechanical ventilation.*

**Comment 2: ‘**The authors mention "Although mortality risk in patients with COVID-19 is tempered by corticosteroids and tocilizumab" however fail to address this in the discussion.

Steroids – (50.9%) 1st wave vs (99.2%) of second wave
Tocilizumab –(6.3%) in 1st wave and (31.8%) of second wave
Remdesivir – (2.9%) in 1st wave and (41.6%) of second wave.?’

**Response 2:** We thank the reviewer for this comment and have made the following changes to the discussion:

We have added the following phrase to the discussion/limitations:

*When we adjust for the key variables in our cox proportional risk analysis model, we find that in the second wave of the pandemic (despite use of repurposed therapeutics such as corticosteroids and Tocilizumab),^13-15^ there is no improvement in ICU mortality. This was particularly surprising considering the benefit of tocilizumab and corticosteroids in reducing COVID-19 mortality is well established.^10,11^*

*It may also be prudent to evaluate whether treatments found to have significant mortality benefit with older COVID-19 variants, continue to be successful as variants mutate and pathophysiology potentially alters.*

***Comment 3: ‘***The discussion still needs improvement based on the above facts*.’*

**Response 3:** Thank you for your comments regarding our discussion and conclusion. We agree and hope your suggested changes have imporeved the manuscript
